# The social gradient in adolescent mental health: mediated or moderated by belief in a just world?

**DOI:** 10.1007/s00787-021-01905-4

**Published:** 2021-11-09

**Authors:** Dominic Weinberg, Gonneke W. J. M. Stevens, Margot Peeters, Kirsten Visser, Jet Tigchelaar, Catrin Finkenauer

**Affiliations:** 1 Department of Interdisciplinary Social Science, Faculty of Social and Behavioural Sciences, Utrecht University, Padualaan 14, 3584 CH Utrecht, The Netherlands; 2 Department of Human Geography and Spatial Planning, Faculty of Geosciences, Utrecht University, Utrecht, The Netherlands; 3 Department of Law, Faculty of Law, Economics and Governance, Utrecht University, Utrecht, The Netherlands

**Keywords:** Social gradient, Adolescent mental health, Socioeconomic status, Health inequalities, Belief in a just world

## Abstract

**Purpose:**

A social gradient in adolescent mental health exists: adolescents with higher socioeconomic status (SES) have fewer mental health problems than their peers with lower SES. Little is known about whether adolescents’ societal beliefs play a role in this social gradient. Belief in a just world (BJW) may be a mediator or moderator of the social gradient in adolescent mental health.

**Methods:**

Using data from 848 adolescents (*M*_age_ = 17) in the Netherlands, path analyses examined whether two indicators of BJW (general and personal) mediated or moderated the associations between two indicators of SES (family affluence and perceived family wealth), and four indicators of adolescent mental health problems (emotional symptoms, conduct problems, hyperactivity, and peer problems).

**Results:**

Adolescents with lower family affluence and lower perceived family wealth reported more emotional symptoms, and the association between perceived family wealth and emotional symptoms was mediated by lower personal and general BJW. Furthermore, higher personal BJW amplified the negative association between SES and peer problems.

**Conclusion:**

This study suggests BJW may both mediate and amplify the social gradient in adolescent mental health. Adolescents’ beliefs about society may be important to include in research aimed at understanding this social gradient.

Many studies across multiple countries have consistently found a social gradient in adolescent mental health: adolescents with lower socioeconomic status (SES) have more mental health problems than adolescents with higher SES [1–3]. However, there are several gaps in the existing knowledge on this topic. First, while most studies show evidence of a gradient, there may be differences in the strength of the gradient across indicators of both SES and mental health. Second, more research is needed into understanding mechanisms that link SES and adolescent mental health—the mediators of the social gradient in adolescent mental health. Third, inconsistency across existing studies regarding the strength of the social gradient in adolescent mental health may be based on the presence of moderators. These three gaps are addressed in this study, through the inclusion of multiple indicators of SES and mental health and by exploring the mediating and moderating potential of adolescents’ beliefs about the world, specifically their Belief in a Just World (BJW). Below, we explain BJW and address why it may be a mediator or a moderator of the social gradient in adolescent mental health.

Differences in the literature regarding the strength of the social gradient in adolescent mental health may be due to the use of different indicators of SES and mental health. Regarding SES, adolescent SES has both objective components—such as markers of parental education, occupation or income—and a subjective component, adolescents’ perception of their family’s SES. Objective SES and subjective SES are proposed to have somewhat different pathways to mental health—objective SES more through the benefits of access to material resources and subjective SES more through psychosocial mechanisms—so it is important that studies of this social gradient include both SES indicators [1, 4]. Regarding mental health, some studies have even found that adolescents with higher SES may show vulnerability for some mental health outcomes (see [5]). Therefore, in this study, we included multiple indicators of SES and of mental health problems.

Adolescence is a period of growing awareness and knowledge of SES, the social context and societal (in)justice [6–9], One important theory regarding how people think about their social context and injustice is Belief in a Just World (BJW, [10]). BJW is the belief that people get what they deserve because the world is fair. This belief is proposed to stem from a fundamental need to believe the world is fair, because this positive illusion acts as a resource that engenders trust, optimism and meaning in people’s lives [10, 11]. While most people to some degree believe that the world is just, there are individual differences in the extent to which people believe this [12]. Research also suggests that there are two separate facets to BJW, distinguished by whether experiences of (in)justice are felt for oneself (personal BJW) or for people in general (general BJW) [11, 13, 14]. BJW also overlaps with several other concepts, including system justification, the psychological motive to defend and justify the status quo [15, 16]. BJW is also a supporting pillar of System Justification Theory [16] and general BJW, in particular, is closely related to system justification [17]. Because both system justification and general BJW capture the extent of belief that people in general are confronted with just systems and institutions [7], for the sake of clarity, in this study we refer to general BJW only. Furthermore, as far as we know, no studies on the social gradient in adolescent mental health have taken into account the role played by BJW in either mediating or moderating this social gradient [1].

Previous research into the mechanisms of the relationship between SES and adolescent mental health have focused mainly on family-level pathways [1]. This research on family-level pathways shows that SES is positively related to the presence of stable and supportive family relationships, and the absence of stressful and threatening environments, and these, in turn, are related to positive adolescent mental health [1, 18, 19]. Only a few studies have considered the role of adolescent beliefs, such as perceptions of personal capability [1]. These suggested mechanisms do not fully explain the social gradient in adolescent mental health; yet, to our knowledge, no research on this social gradient has explored the mediational role of adolescents’ beliefs about society. Yet there are reasons to think that BJW may mediate the social gradient in adolescent mental health, because theories and evidence suggest that SES may be associated with BJW, and BJW, in turn, associated with adolescent mental health. Regarding the link between SES and BJW; compared to their peers with higher SES, adolescents with lower SES face greater adversity and instability [20]. Evidence suggests these adolescents may therefore have lower BJW because they are likely to have experienced more injustice, or they may perceive society to be more unjust as a way to understand their lower SES position in society [7, 21]. Few empirical studies have examined this association in adolescents, but those that do have shown that Kenyan, Brazilian and Chinese adolescents with lower SES have lower BJW [22–24]. We expect that the relationship between SES and BJW may be stronger for subjective SES than for objective SES given that subjective SES and BJW are both based on adolescents’ perceptions of their place in the world. In turn, adolescents with lower BJW may doubt that the social context is just and meaningful, may find it difficult to consider people to be trustworthy, and they may be less optimistic about their life chances, making them more susceptible to mental health problems [7, 25]. Indeed, there is robust empirical evidence that adolescents with lower BJW have more mental health problems [7, 12, 26]. These findings suggest BJW may be a mediator of the social gradient in adolescent mental health, and this may be especially likely to be the case for subjective SES.

Furthermore, there are also reasons to believe BJW may moderate the social gradient, either by weakening or strengthening it. On the one hand, *higher* BJW may weaken the relationship between SES and adolescent mental health. Adolescents with higher BJW may feel more in control of their own fate and capable of achieving their goals [27–29] and these feelings may be important mechanisms that buffer against stresses more typically associated with being lower SES [30, 31]. There is evidence that BJW weakens the link between SES indicators (such as unemployment and poverty) and mental health [32, 33], although no studies to date have specifically looked at BJW as a buffer of the social gradient in adolescent mental health. On the other hand, *higher* BJW may instead amplify the social gradient in adolescent mental health. Adolescents with higher BJW are more likely to attribute outcomes to internal factors, such as effort and ability, rather than to external factors, such as structural privileges and barriers [34–36]. In consequence, adolescents with higher BJW may believe that their (family) SES is based on talent and effort, generating self-enhancing feelings of pride in adolescents with higher SES, and self-debilitating feelings of shame and inferiority in adolescents with lower SES [37–39]. This suggests mental health differences across the SES spectrum may be heightened for adolescents with higher BJW.

In sum, in this study, we investigated whether there was a social gradient in mental health in adolescents in the Netherlands. As discussed above, adolescent SES has both objective and subjective components, therefore we included measures of family affluence (objective SES) and perceived family wealth (subjective SES) [1, 4]. We included measures of both personal and general BJW. We studied four indicators of mental health problems, as measured by the strengths and difficulties questionnaire: emotional symptoms, conduct problems, hyperactivity, and peer problems [40]. We hypothesised that the gradient would be mediated by BJW (both personal and general) and also explored a potential moderating effect of personal and general BJW.

## Methods

### Sample

We used data from the first wave of the ongoing longitudinal YOUth Got Talent project on the SES-health gradient in adolescence (*N* = 1231). Data were collected between September 2019 and February 2020 from adolescents at three vocational schools in the Utrecht region of the Netherlands. Classes were selected within these schools, covering the fields of creative, technical and health education. The adolescent response rate was 81%: non-participating students were sick or absent from the classroom (15%); chose not to fill in the questionnaire or refused to participate (3%); or gave consistently invalid responses (2%). Self-report questionnaires (digital for 96.5% and paper-and-pencil for 3.5%) were administered in the classroom, taking roughly 20–30 min. Tertiary vocational schools in the Netherlands are divided into four levels (1—entry-level; 2—basic, 3—professional; 4—middle-management) [41]. Pilot research revealed that adolescents in Level 1 classes were unable to complete the questionnaire satisfactorily, so they were not included in this study. Based on researcher expectations that students in lower levels would have lower attention levels, students in Levels 2 and 3 (*n* = 322) completed a shortened questionnaire, which did not include the personal BJW scale. The analysis sample for this study was thus students in Level 4 only (*n* = 909). After taking into account differences between classes in the covariates (gender, age and migration background), the class-level ICC for all main study variables (SES, BJW and mental health variables) was small (i.e., < 10% of the variance in the main variables was at the class level, see [42]). For most of these variables, the ICC was negligible (< 2.5%), so we determined that adjusting for clustering in this study was unnecessary.

Participants gave active consent and were informed that data would be anonymised. Students in Level 4 were included in the analyses if they had data on all SES and control variables and reported at least one BJW score and one mental health outcome (*n* = 848, 93.3% of the sample). Roughly half of the participants were female (54%) and 12% had a non-western migration background. Compared with excluded adolescents, included adolescents were less likely to have a non-western migration background (12 vs 30%; *χ*^2^(1) = 13.93, *p* < 0.001), but this difference was small [43]. There were no differences between included and excluded adolescents on the SES or other control variables. Ethical approval was gained from the Ethics Assessment Committee of the Faculty of Social Sciences at Utrecht University (FETC18-070) in 2018.

### Measures

#### Socioeconomic status

*Family affluence* was measured using the six-item Family Affluence Scale (FAS), which assessed family material assets: car/van ownership, having own bedroom, holidays abroad, computer ownership, dishwasher ownership, and bathrooms [44]. Item scores were summed to compute an ordinal score for participants who completed all scale items, then this ordinal score was ridit-transformed into a continuous family affluence score (range = 0–1; mean = 0.5), with a higher score indicating more material assets [45]. The FAS is a reliable and valid instrument that enables adolescent to report their family affluence [44].

*Perceived family wealth* was measured with the question, ‘How well off do you think your family is’? The item had a 5-point response scale from 1 (*very well off*) to 5 (*not at all well off*), which was reversed so that higher scores indicated higher perceived family wealth. The measure has been found to be easy to answer for adolescents [46].

#### Belief in a just world

*Personal BJW* was measured using the personal belief in a just world scale [47]. Seven items measured the belief that events in one’s life are just (e.g., ‘I believe that I usually get what I deserve’, ‘I am usually treated fairly’) with a 7-point Likert scale ranging from 1 (*totally disagree*) to 7 (*totally agree*). An average score was computed for respondents who answered more than half of the items; a higher score indicated a stronger personal BJW (Cronbach’s *α* = 0.81).

*General BJW* was measured using an adaptation of the system justification scale [34, 48]. Eleven items measured the belief that the Dutch socio-political and economic system is just (e.g., ‘In general, Dutch society is fair, ‘People get fair treatment in the Netherlands, no matter who they are’) with a 7-point Likert scale ranging from 1 (*totally disagree*) to 7 (*totally agree*). An average score was computed for respondents who answered more than half of the items; a higher score indicated a stronger general BJW (*α* = 0.89).

#### Adolescent mental health problems

*Emotional symptoms*, *conduct problems*, *hyperactivity* and *peer problems* were measured with the SDQ-R: a revised version of the Strengths and Difficulties Questionnaire (SDQ), which has better psychometric properties for the problem subscales than the original self-report SDQ [40, 49]. The SDQ asks about behaviour and feelings over the past 6 months with a 3-point ordinal response scale: 0 (*not true*), 1 (*somewhat true*), 2 (*certainly true*). Examples of items include ﻿‘I get very angry and often lose my temper’ and “I worry a lot”. The SDQ-R consists of 15 items measuring 4 subscales—emotional symptoms (5 items), conduct problems (4 items), hyperactivity–inattention problems (3 items) and peer relationship problems (3 items). Two subscales, emotional symptoms (ordinal *α* = 0.82) and hyperactivity–inattention problems (*α* = 0.79), had good internal consistency (ordinal *α* > 0.70) [50], though internal consistency for conduct problems (*α* = 0.59) and peer problems subscales (*α* = 0.52) was less good, in line with former research [49]. For participants who completed more than half of the subscale items, we computed mean scores, which were then multiplied by five to retain comparability with the original SDQ, with higher subscale scores indicating more problems (ranging from 0 to 10).

#### Control variables

We controlled for gender, age and migration background given their effect on adolescent mental health in the Netherlands (Duinhof et al., 2020). We asked whether the participant was a girl (coded 0) or boy (coded 1). We asked about month and year of birth to calculate age at the date of data collection. Conforming with previous research in the Netherlands, and Dutch statistical agencies, migration status was measured by asking adolescents about their parents' countries of birth. We distinguished between: adolescents with both parents born in the Netherlands, adolescents with at least one parent with a western immigration background, and adolescents with at least one parent with a non-western immigration background [51, 52]. Only 5.9% of adolescents had a western immigration background, so this group was merged with adolescents whose parents were born in the Netherlands, given both groups are western.

### Data analysis

**Fig. 1:**
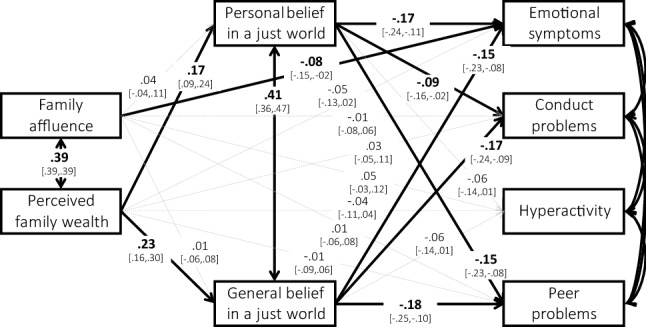
The association between SES and adolescent mental health problems mediated by BJW (Model 2). Standardised coefficients. Continuous lines and bold numbers indicate significant paths (*p* < 0.05); dotted lines and regular numbers indicate insignificant paths (*p* > 0.05). Results for control variables (gender, age, and migration status) and total and indirect effects shown in Table 2. Model fit: *χ*^2^ (6) = 21.41, *p* < 0.001, CFI = 0.981, RMSEA = 0.055

To test pathways between SES, BJW and mental health, relationships between the variables were examined with structural equation modelling (SEM), using the R package lavaan [53]. Missing data were modelled using the default Maximum Likelihood estimator. Goodness-of-fit was evaluated using two measures, with good model fit indicated by CFI ≥ 0.95 and RMSEA < 0.05 [54]. We first specified a mediation model (1), by regressing the control variables (gender, age and migration status) and the two SES indicators (family affluence and perceived family wealth) on the four mental health outcomes, including both personal BJW and general BJW as mediators of the pathways between SES and mental health (see Fig. 1). The model included a bidirectional pathway between the two BJW indicators, given they were expected to correlate [13]. Next, to explore whether BJW moderated pathways between SES and mental health, we regressed the control variables, both SES indicators, both BJW indicators and, one at a time, interaction terms between the two SES and BJW indicators on mental health (Models 3a-d). Evidence for mediational pathways was established based on the direct effects, indirect effects (estimated by lavaan) and summing these to compute total effects [53]. Independent variables were considered fixed, so their means, variances and covariances were fixed to sample values [53]. We used an *α* level of 0.05, and to control for inflation of Type I error rates based on multiple testing, we applied the Benjamini–Hochberg procedure with a false discovery rate of 0.05 [55]. We interpreted standardised regression coefficients as negligible (|*r*|< 0.1), small (|*r*|= 0.1–0.3), medium (|*r*|= 0.3–0.5), or large (|*r*|> 0.5) [43]. Given our sample size (*n* = 848), we had sufficient statistical power to detect effects in models with fewer than 43 parameters, based on the 20:1 ratio [56]. The preregistered analysis plan, as well as analysis scripts, can be found at https://osf.io/js3em/.^1^

## Results

### Descriptive statistics

Table 1 shows variable means, standard deviations and correlations between the variables. Compared to the scale midpoints, adolescents’ perceptions of their family wealth were average, they reported quite high personal BJW and general BJW, and they reported low levels of mental health problems. The SES indicators (family affluence and perceived family wealth) had medium positive associations with each other (*r* = 0.39), as did the BJW indicators (*r* = 0.44), and there were significant small positive associations between the SES indicators and BJW indicators (*r*s range from 0.10 to 0.23). Mental health problems had small to medium positive associations with each other (*r*s range from 0.08 to 0.35) and negligible to medium negative associations with SES and BJW indicators (*r*s range from − 0.02 to − 0.30), except for the association between family affluence and hyperactivity (*r* = 0.03).

**Table 1:** Descriptives and correlation table (*n* = 848)

	1	2	3	4	5	6	7	8	9	10	11
1. Age											
2. Gender^a^	.04										
3. Migration background^b^	.10**	− .01									
4. Family affluence	− .15**	.04	− .17**								
5. Perceived family wealth	− .13**	.09*	− .21**	.39**							
6. Personal BJW	− .05	.16**	− .05	.10**	.18**						
7. General BJW	− .05	.04	− .03	.10**	.23**	.44**					
8. Emotional symptoms	.10**	− .33**	.00	− .15**	− .17**	− .30**	− .26**				
9. Conduct problems	.00	.08*	.01	− .02	− .02	− .14**	− .20**	.24**			
10. Hyperactivity	− .05	− .06	− .04	.03	− .04	− .10**	− .10**	.28**	.28**		
11. Peer problems	.14**	− .04	.03	− .05	− .10**	− .24**	− .25**	.35**	.21**	.08*	
Mean	17.14			0.53	3.06	4.64	4.34	3.28	0.92	4.69	2.61
Standard Deviation	1.54			0.28	0.72	0.90	0.98	2.54	1.33	3.00	1.94
Range	16–26			0–1	1–5	1–7	1–6.64	0–10	0–8.75	0–10	0–10

* *p* < .05. ** *p* < .01

^a^Reference category: girl

^b^Reference category: Dutch/western

Table 2 and Fig. 1 show the results of Model 1, investigating mediation. Regarding the control variables, older adolescents had slightly higher levels of emotional symptoms and peer problems, girls had higher levels of emotional symptoms and negligibly lower levels of conduct problems than boys, and there were no differences in mental health problems based on migration background. Regarding the social gradient, there were small negative total effects of both family affluence and perceived family wealth on emotional symptoms (see Table 2).

**Table 2:** SEMs investigating mediation and moderation of the social gradient in adolescent mental health by BJW

Model		Emotional symptoms		Conduct problems		Hyperactivity		Peer problems	
		*β*	*CI*	*β*	*CI*	*β*	*CI*	*β*	*CI*
1	Age	.08*	[.01,.14]	− .01	[− .09,.06]	− .05	[− .12,.02]	.12**	[.06,.19]
	Gender^a^	− .29**	[− .35,− .24]	.10**	[.03,.17]	− .05	[− .12,.02]	− .01	[− .08,.05]
	Migr. background^b^	.05	[− .02,.12]	− .01	[− .07,.06]	.04	[− .03,.11]	.00	[− .07,.07]
	FA (total)	− .09*	[− .16,− .02]	− .02	[− .09,.05]	.04	[− .03,.12]	.00	[− .07,.08]
	FA (indirect through Personal BJW)	− .01	[− .02,.01]	.00	[− .01,.00]	.00	[− .01,.00]	− .01	[− .02,.01]
	FA (indirect through General BJW)	.00	[− .01,.01]	.00	[− .01,.01]	.00	[− .01,.00]	.00	[− .01,.01]
	PFW (total)	− .12**	[− .19,− .04]	− .02	[− .10,.06]	− .06	[− .14,.01]	− .08 ^c^	[− .16,.00]
	PFW (indirect through Personal BJW)	− .03**	[− .05,− .01]	− .02 ^c^	[− .03,.00]	− .01	[− .02,.00]	− .03**	[− .04,− .01]
	PFW (indirect through General BJW)	− .04**	[− .06,− .02]	− .04**	[− .06,− .02]	− .01	[− .03,.00]	− .04**	[− .06,− .02]
2a	FA * Personal BJW	.01	[− .34,.37]	− .27	[− .58,.05]	.05	[− .33,.43]	− .45 ^c^	[− .86,− .04]
2b	PFW * Personal BJW	− .36	[− .82,.10]	− .17	[− .62,.28]	− .31	[− .74,.11]	− .47*	[− .84,− .11]
2c	FA * General BJW	.16	[− .16,.48]	− .16	[− .49,.18]	.10	[− .28,.48]	− .40 ^c^	[− .75,− .05]
2d	PFW * General BJW	.00	[− .40,.40]	− .27	[− .75,.21]	− .05	[− .48,.38]	− .24	[− .64,.16]

*Migr.* Migration, *FA* family affluence, *PFW* perceived family wealth, *BJW* belief in a just world

*p < .05. **p < .01. All models (except model 2, see Fig. 1) were fully saturated: *df* = 0, so CFI = 1, RMSEA = 0

^a^Reference category: girl

^b^Reference category: Dutch/western

^c^Parameter insignificant after applying Benjamini–Hochberg procedure. Results for main mediation pathways are shown in Fig. 1

Regarding the mediation model, the total effect of both family affluence and perceived family wealth on emotional symptoms was negative, indicating a social gradient in emotional symptoms by both SES indicators. Family affluence was also directly negatively associated with emotional symptoms, but there were no other direct effects of the SES indicators on mental health problems. Regarding mediation pathways through BJW, family affluence was not associated with personal BJW and general BJW, so there were no mediation effects of family affluence. However, perceived family wealth was positively associated with both personal BJW and general BJW, which were both, in turn, negatively associated with emotional symptoms, conduct problems and peer problems, but not hyperactivity. Significant indirect effects were found for perceived family wealth on emotional symptoms and peer problems through both personal BJW and general BJW, and for conduct problems through general BJW only. In sum, for perceived family wealth, the model indicated full mediation for emotional symptoms (total and indirect effects, and no direct effect), and indirect only effects for conduct problems and peer problems (i.e., no direct effect).

**Fig. 2:**
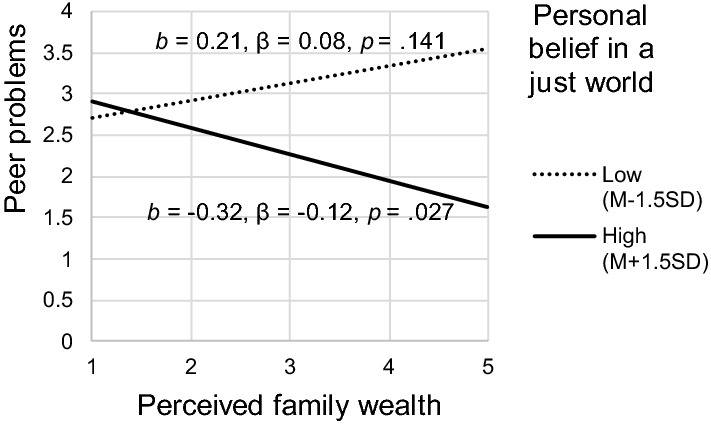
Conditional effects of perceived family wealth on peer problems for two values of personal BJW. (Age, Family affluence, and General BJW at mean values, Gender and Migration background at reference group values, i.e., Dutch/western girls)

The moderation models (2a–d) showed that the association between perceived family wealth and peer problems was moderated by personal BJW. To facilitate interpretation, we depicted this association for two values of personal BJW: low (one and a half standard deviations below the mean) and high (one and a half standard deviations above the mean; Fig. 2). Among adolescents with low personal BJW, perceived family wealth was not associated with peer problems, while, among adolescents with high personal BJW, perceived family wealth was negatively associated with peer problems. Thus, high personal BJW amplified the association between perceived family wealth and peer problems.

## Discussion

To our knowledge, this study is the first to investigate the role of individual-level BJW in the social gradient in adolescent mental health. Firstly, we found a social gradient for one of four mental health outcomes, whereby adolescents with lower family affluence and adolescents with lower perceived family wealth reported more emotional symptoms. Secondly, the latter association was mediated by BJW, such that adolescents with lower perceived family wealth had lower personal and general BJW, which in turn were related to more emotional symptoms. For two other indicators of mental health problems, peer problems and conduct problems, though perceived family wealth had no direct association with these mental health problems, there was an indirect effect, such that adolescents with lower perceived family wealth had lower general BJW, which in turn was associated with more peer problems and conduct problems. For peer problems, there was also an equivalent indirect effect through personal BJW. Thirdly, there was some evidence of a moderating effect of personal BJW. Adolescents with higher perceived family wealth reported fewer peer problems, which was increasingly true at higher levels of personal BJW.

The results suggest that there is a social gradient in emotional symptoms for both family affluence and perceived family wealth, which replicates previous results in the Netherlands, and other countries with relatively high standards of living [57, 58]. There was also a negative univariate association between perceived family wealth and peer problems, though this attenuated to become insignificant in the mediation model with both SES indicators, implying overlap in the mechanisms linking these two SES indicators and adolescent mental health [4].

In our mediation model adolescents with lower perceived family wealth had lower personal and general BJW, while family affluence was not associated with either BJW indicator. One explanation for these differential results may be that perceived family wealth more strongly represents social position compared to others than family affluence does. Adolescents who perceive that their family is less well off than other families may feel this is unjust or doubt whether the world can be fair [7, 21]. Our research suggests that not only in countries like Kenya, Brazil and China [22–24], but also in a country with a relatively high standard of living such as the Netherlands, adolescents with lower perceived family wealth see the world as less just than their higher SES peers. Furthermore, as expected, adolescents with lower BJW had higher levels of emotional symptoms, conduct problems, and peer problems. Existing research suggests that adolescents with lower BJW may be less trusting of others and less able to cope with stressful experiences [7]. Just World Theory also suggests that these adolescents may be less optimistic and feel that their lives are less meaningful, and empirical evidence suggests adolescents with lower BJW may have more mental health problems because they feel less control over their own fate and less capable of achieving their goals [27–29]. Both personal BJW and general BJW were independently associated with adolescent mental health problems, supporting findings that these two constructs have somewhat different pathways to mental health [13, 14].

We also found some evidence that BJW moderated the social gradient in adolescent mental health: higher personal BJW amplified associations between perceived family wealth and peer problems. Adolescents with higher personal BJW may believe that because the world is fair and people get what they deserve, that their SES is based on talent and effort [34–36], intensifying the relationship between SES and feelings of pride (versus shame) about their social status [37–39]. This finding may apply specifically to peer problems because status plays a central role in the task of building relationships with peers [59]. However, given that we found no evidence of moderation for three other indicators of mental health problems, the evidence for moderation seems relatively weaker than that for mediation. This study had several strengths, in particular the integration of BJW into research on the social gradient in adolescent mental health. Additionally, we used multiple indicators of the key concepts and included over 1,000 adolescents from a sample of students in vocational schools, a group typically under-represented in existing research [60]. The study also has several limitations. Firstly, the cross-sectional nature of our data restricts us from drawing causal conclusions. Poor mental health may instead lead to lower BJW, or a third variable, such as optimism, may explain the association between perceived family wealth, BJW, and mental health. Secondly, the Youth Got Talent dataset is limited to adolescents in one type of school (vocational) in one region in the Netherlands. In our analyses, we only included adolescents in the middle-management educational level (Level 4), and furthermore, adolescents with a non-western migration background had a higher likelihood of being excluded from the sample, so these results may not generalise to adolescents in other educational levels or in samples with more diversity in migration background. Associations between adolescent SES and beliefs about the world may depend on adolescents’ educational and occupational opportunities [61–63], so further research could investigate if our findings generalise to adolescents in a broader range of educational tracks, adolescents in more rural areas and adolescents in other countries. Thirdly, several measures had limitations. To measure general BJW, we used the system justification scale, which, compared to other scales used to measure general BJW, is focussed more strongly on the (in)justice of systems and institutions, rather than the world more broadly. The measure may be less suited to capturing those for whom BJW is based on belief that other causal forces, such as God, or nature, determine whether people get what they deserve [64]. Participants may not have interpreted our single-item measure of perceived family wealth as a social comparison, because it did not explicitly include a comparative frame. Furthermore, this item is limited by being a Likert-item, which provides an ordinal approximation of a continuous variable. However, a review of research on the association between subjective SES and adolescent mental health found that Likert scales have similar associations with adolescent health outcomes as other measures [4].

Future research and practice could address several other issues arising from this study. Firstly, longitudinal research would help with identifying causal pathways in the development of the social gradient in adolescent mental health. Secondly, given beliefs, such as BJW, system justification, and meritocracy (people get what they deserve because the system rewards individual talent and effort) are closely related [15], future research could try to further disentangle which are the key mechanisms that affect the social gradient. One previous study has investigated meritocratic beliefs [51], but, rather than looking at individual-level meritocratic beliefs, it focussed on whether aggregated meritocratic beliefs at the country-level explained cross-country differences in the strength of the social gradient. For example, future research could explore the extent to which adolescents attribute their SES to internal or external causes, and whether this relates to their BJW and/or their feelings of pride or shame regarding their SES [65]. Thirdly, research could unpick the mechanisms by which adolescents' direct experiences of (in)justice, and their wider knowledge and perceptions of (in)justice in the world, contribute to adolescent mental health [13, 22, 66]. Fourthly, interventions to reduce the social gradient in adolescent mental could consider adolescents beliefs about society, although specific suggestions for practice seem premature given that BJW both mediates and amplifies the social gradient.

Overall, our findings suggest that BJW can contribute to our understanding of the social gradient in adolescent mental health. In particular, we found that BJW (both personal and general) mediated the association between perceived family and emotional symptoms. Also, higher personal BJW was found to amplify the association between perceived family wealth and adolescent peer problems. This study should be seen as a first step in understanding the role of adolescents’ beliefs about society in the social gradient in adolescent mental health, and our findings suggest that further exploration of these ideas would be a fruitful and important avenue for research.

## Data Availability

The data are not currently publicly available because it comes from an ongoing longitudinal study (Youth Got Talent). Collaborators who are interested in using the data are welcome to contact the corresponding author to discuss access to the data.

## Footnotes

1 Two changes were made to the preregistered analysis plan. Second, we preregistered a fourth model which included both moderation and mediation pathways, but subsequently decided such a model was conceptually unnecessary for answering the proposed research questions. Third, following the helpful suggestions of the reviewers, we did not include participants in Levels 2 and 3 in the analysed sample.

## Code availability

The data analysis scripts, the codebook for the Youth Got Talent project, and a preregistered data analysis plan are available on the Open Science Framework (https://osf.io/js3em/).
